# Unveiling the Anti-Angiogenic Potential of Small-Molecule (Kinase) Inhibitors for Application in Rheumatoid Arthritis

**DOI:** 10.3390/cells14020102

**Published:** 2025-01-11

**Authors:** Fatemeh Khodadust, Eva M. L. Philippon, Maarten M. Steinz, Jan Piet van Hamburg, Johan van Meerloo, Judy R. van Beijnum, Gerrit Jansen, Sander W. Tas, Conny J. van der Laken

**Affiliations:** 1 Department of Rheumatology & Clinical Immunology, Amsterdam UMC, Meibergdreef 9, 1105 AZ Amsterdam, The Netherlands; f.khodadustvaskasi@amsterdamumc.nl (F.K.); s.w.tas@amsterdamumc.nl (S.W.T.); 2 Department of Experimental Immunology, Amsterdam UMC, Meibergdreef 9, 1105 AZ Amsterdam, The Netherlands; 3 Department of Hematology, Amsterdam UMC, De Boelelaan 1117, 1081 HV Amsterdam, The Netherlands; 4 Cancer Center Amsterdam, Amsterdam UMC, 1081 HV Amsterdam, The Netherlands; 5 Angiogenesis Laboratory, Department of Medical Oncology, Amsterdam UMC, De Boelelaan 1117, 1081 HV Amsterdam, The Netherlands

**Keywords:** angiogenesis, rheumatoid arthritis, 3D model, spheroid, small-molecule inhibitors, endothelial cells, synovial inflammation

## Abstract

Rheumatoid arthritis (RA) is a chronic autoimmune disease characterized by inflammation leading to joint damage and systemic complications. Angiogenesis promotes inflammation and contributes to RA progression. This study evaluated potential anti-angiogenic effects of several compounds including small-molecule kinase inhibitors, such as sunitinib (pan-kinase inhibitor), tofacitinib (JAK-inhibitor), NIKi (NF-κB-inducing kinase inhibitor), and the integrin-targeting peptide fluciclatide, using a scratch assay and 3D spheroid-based models of angiogenesis. For all drugs tested in the low micromolar range (1–25 μM), sunitinib (as positive anti-angiogenetic control) showed marked inhibition of endothelial cell (EC) migration and sprouting, effectively reducing both scratch closure and sprout formation. Tofacitinib exhibited marginal effectiveness in the scratch assay, but performed better in the 3D models (55% inhibition), whereas NIKi showed around 50% anti-angiogenic effects in both models. Fluciclatide changed EC morphology rather than migration, and only when stimulated with synovial fluid in spheroid model did it show inhibitory effects (at ≥2.5 µM), with none below this dosage. These results highlight the potential of NIKi and tofacitinib for angiogenesis inhibition and of fluciclatide for safe diagnostic targeting of microdose in RA, as well as the need for advanced screening models that mimic RA’s complex inflammatory pro-angiogenic environment.

## 1. Introduction

Rheumatoid arthritis (RA) is a chronic inflammatory autoimmune disease which primarily affects the synovial joints and leads to progressive joint and bone destruction, disability, and systemic complications [1,2,3]. RA develops in several stages, with a preclinical phase during which a combination of genetic and environmental factors causes immune activation and the production of autoantibodies, including anticitrullinated protein antibodies (ACPA) or rheumatoid factor (RF) [4,5]. Yet, some individuals may be seronegative for these autoantibodies. In this phase, individuals may show arthralgia without obvious inflammation [4]. The transition to established RA involves subclinical inflammation, vascular changes, and, ultimately, clinically evident arthritis [4,6].

Angiogenesis, the development of new blood vessels from the pre-existing vascular network, is one of the key processes in the pathogenesis of RA, even in its earliest phases [6,7,8]. Synovial tissue of RA patients exhibits increased vascularity and angiogenic activity [9]. Chronic inflammation, driven by immune cells infiltrating the synovium, along with the growing need for oxygen and nutrients of proliferating cells within the inflamed tissue, are the main causes [10]. In addition, numerous factors have been found to impact vascular development and morphology, including hypoxia, pro-inflammatory cytokines, and mechanical factors such as shear stress [7,8,11]. As such, the process of angiogenesis offers opportunities for specific therapeutic targeting, as well as disease (response) monitoring with molecular imaging techniques [12,13,14,15,16]. This may be of additive value to available RA treatment with conventional synthetic disease-modifying anti-rheumatic drugs (DMARDs), such as methotrexate and hydroxychloroquine, and/or biological DMARDs like adalimumab (anti-TNFα) or tocilizumab (anti-IL-6R), which still face inadequate responses in part of the RA patients [17]. In such cases, targeted synthetic DMRDs, like tofacitinib and baricitinib (Janus Kinase inhibitors), offer an alternative approach [17]. Given that RA is incurable, considering adjuvant therapies could improve overall treatment outcomes [13,17]. Further research exploring novel treatment strategies, including targeting of angiogenesis, is essential for the management of RA [13].

Targeting the intricate process of angiogenesis in RA requires a thorough understanding of the disease and the development of innovative treatment and diagnostic approaches. Vascular endothelial growth factor (VEGF) inhibitors, fibroblast growth factor (FGF) inhibitors, and angiopoietin-2 inhibitors are examples of anti-angiogenic agents that are currently being used as potential strategies for angiogenesis inhibition therapy in RA [16,18,19,20]. These agents have all demonstrated efficacy in reducing inflammation, attenuating pathological neovascularization, and ameliorating joint destruction in RA animal models and clinical trials [21,22,23]. Along with these studies, molecular imaging with positron emission tomography (PET), with its ability to visualize and assess biological processes at a molecular level, has proved to be of supportive value in monitoring of RA disease activity and may also be a valuable tool to monitor the anti-angiogenetic effects of potential RA drugs [15,24,25].

Anti-angiogenesis-based therapy for RA is still in its experimental stage [13,16], and further research is required in order to establish its efficacy and safety of established and novel drugs. To this end, relevant in vitro models mimicking in vivo angiogenesis are helpful to screen and identify candidate drugs with anti-angiogenic therapeutic effects and/or angiogenesis diagnostics. Conventional in vitro models frequently only include endothelial cells (EC). However, the addition of other relevant cell types, such as stromal and immune cells, could make these models even more relevant to the intricate pathological angiogenesis seen in RA [26]. We recently developed a three-dimensional (3D) co-culture model to more accurately represent the complicated nature of angiogenesis in RA. Two complementary variants of this model were developed that enable a more comprehensive analysis of angiogenesis [26].

In the first model, normal human dermal fibroblasts (NHDFs) and primary human umbilical vein endothelial cells (HUVECs) are combined and treated with growth factors within a collagen matrix to mimic general angiogenesis. This model lacks the intricacy of RA-specific microenvironments, and is straightforward for screening purposes. Our second model, on the other hand, better replicates the complex cellular interactions in the RA synovial tissue by incorporating RA fibroblast-like synoviocytes (FLS) and HUVEC in the presence of RA synovial fluid (SF). By offering a more accurate representation of aberrant angiogenesis in RA, this model may allow for a deeper understanding of the anti-angiogenic potential of therapeutic drugs in the context of RA, where both angiogenesis and inflammation are key factors. In this study, our primary objective was to assess the anti-angiogenic properties of four compounds with distinct mechanisms of action.

Using two screening models—the scratch assay [27] and the spheroid-based models [26]—we sought to investigate the drugs’ efficacy in inhibiting EC functions, evaluate assay sensitivity, and ascertain the suitability of both assays for the comparative analysis of anti-angiogenic drug candidates. We tested the following small molecule (kinase) inhibitors: (i) sunitinib, a pan-receptor tyrosine kinase inhibitor with strong affinity for the VEGFR family as a positive control [22,28]; (ii) tofacitinib, an inhibitor of Janus kinases 1 and 3 (JAK1/3) with proposed anti-angiogenic effects apart from its anti-inflammatory properties in RA [29,30,31]; (iii) a small-molecule NF-κB-inducing kinase inhibitor (NIKi) which has shown potential in both inhibiting pathological angiogenesis and suppressing inflammation in RA [26,32]; and (iv) fluciclatide, a small synthetic cyclic peptide containing an RGD-sequence (Arg-Gly-Asp) motif that selectively targets the α_v_β_3_/α_v_β_5_ integrin receptor being upregulated on vascular ECs [33]. While fluciclatide is primarily explored as a promising molecular imaging agent for PET ([^18^F]-Fluciclatide) [34,35], we also investigated its potential biological activity in suppressing angiogenesis, which is essential for determination of a safe diagnostic dose. By comparing these compounds in two complementary in vitro models, we generate novel insight into their relative efficacy, demonstrate the importance of preclinical models in evaluating therapies targeting angiogenesis, and propose fluciclatide as a candidate for dual diagnostic and therapeutic (theranostic) application in RA.

## 2. Materials and Methods

### 2.1. Chemicals/Drugs

Sunitinib and tofacitinib citrate (both from SelleckChem, Houston, TX, USA) were dissolved in DMSO as 10 mM and 25 mM stock solutions, respectively. Fluciclatide (GE Healthcare, Little Chalfont, UK) and NIKi (AstraZeneca, Gothenburg, Sweden) were also dissolved in DMSO as a 10 mM stock solution. All compounds were stored at −20 °C. The chemical structures are shown in Figure S1.

### 2.2. Cell Culture

HMEC-1 (CRL-3243; ATCC) [36] cells were maintained in RPMI-1640 cell culture medium supplemented with 10% newborn calf serum (NBCS) (Sigma-Aldrich, St. Louis, MI, USA), 10% human serum [37], 2 mM L-glutamine (Gibco, Carlsbad, CA, USA), and 1% of antibiotics (penicillin/streptomycin, Life Technologies, Carlsbad, CA, USA). HUVEC cells were harvested from the human umbilical veins of healthy volunteers (3 donors). Primary HUVEC cells were cultured in culture flasks coated with 1% gelatin (Sigma-Aldrich, St. Louis, MI, USA) in M199 (Gibco, Carlsbad, CA, USA) containing 20% fetal calf serum (FCS) (Biowest, Q13 Ennigerloh, Germany), 100 mg/mL penicillin/streptomycin (Gibco, Carlsbad, CA, USA), 50 mg/mL heparin (Sigma-Aldrich, St. Louis, MI, USA), 12.5 mg/mL endothelial cell growth supplement (ECGS) (Corning, NY, USA), and 2 mM L-glutamine and expanded until passage 4. RAFLS were sourced from RA patients following the method outlined in previous studies [38]. A mixture of synovial fluid was prepared by combining equal volume of samples from 13 RA patients. RAFLS and NHDF were cultured in DMEM (Gibco, Carlsbad, CA, USA) supplemented with 10% FCS (Biowest, Ennigerloh, Germany), 100 mg/mL penicillin/streptomycin, 2 mM L-glutamine (Gibco, Carlsbad, CA, USA), 10 mM HEPES (Gibco, Carlsbad, CA, USA), and 250 mg/mL gentamicin (Gibco, Carlsbad, CA, USA) up to passage 9. For patient materials, all participants provided written consent in accordance with the Declaration of Helsinki, and the study received approval from the Medical Ethics Committee at the Academic Medical Centre, University of Amsterdam, The Netherlands.

### 2.3. Scratch Assay

A total of 5000 HMEC-1 cells/well were seeded in a flat-bottom 96-well culture plate (VWR) pre-coated with 0.2% gelatin/PBS for 30 min. After 48 h incubation, at ∼80% cell confluence, scratches (approximately 400 µm wide) were made using a 96-pin wound maker (Peira Scientific Instruments, Belgium), after which the media were replaced with fresh complete media. Baseline images (t = 0) were taken. Drugs at specific concentration ranges were subsequently added to the wells (100 µL/well), followed by incubation at 37 °C in a humidified 5% CO_2_ atmosphere (and for NIKi experiments, LIGHT (TNFSF14) (100 ng/mL) was added 2 h after addition of NIKi to the medium to stimulate non-canonical NF-κB signaling). Images of scratch closures were taken at t = 4, 6, 8, 16, and 24 h. Readouts were considered as the extent of scratch closure or inhibition of cell migration observed at specific time points. All images were captured on an automated platform using a Leica DMI3000B microscope (Leica, Rijswijk, The Netherlands) at 5× magnification and a Pulnix RMC1327GE camera under the control of Universal Grab software (DCILabs, Keerbergen, Belgium). Scratch areas (µm^2^) were quantified using automated Scratch Analysis software (ScratchAssay 6.2 (DCIlabs)) [39,40]. For each experimental condition, comparisons between each drug concentration and the control group were performed using paired *t*-tests at multiple time points (8, 16, and 24 h). The percentage of wound closure was calculated relative to the baseline.

### 2.4. 3D Spheroid-Based Models of Angiogenesis

The workflow of the 3D model and its quantitative image analysis are depicted in Figure S2. In detail, spheroid co-cultures were performed in EC growth medium-2 (EGM-2) (Lonza, Basel, Switzerland) supplemented with 2% FCS (*v*/*v*), hydrocortisone, epithelial growth factor (EGF), insulin-like growth factor-1, ascorbic acid, GA-100, heparin, basic fibroblast growth factor (bFGF), and VEGF (PeproTech, Thermo Fisher, Waltham, MA, USA).

To prepare a 1.2% (*w*/*v*) methocel solution, 6 g of methylcellulose (Sigma-Aldrich, St. Louis, MI, USA) was autoclaved and dissolved in 500 mL of M199 medium (Gibco, Carlsbad, CA, USA). The cells, grown to approximately 80% confluency, were incubated in a 2 mM solution of either CellTracker Orange CMRA or CellTracker Green CMFDA in EGM-2 medium (both from Molecular Probes/Invitrogen, Paisley, UK) for 30 min at 37 °C in a CO₂ incubator, and subsequently harvested and counted. A total of 7.5 × 10^4^ HUVEC and 3.75 × 10^4^ NHDF or RAFLS cells were pooled together and suspended in 15 mL of 20% methocel solution (*v*/*v*) in EGM-2. This cell mixture was then distributed in 150 µL volumes into a 96 U-well suspension plate (Greiner BioOne, Stonehouse, UK). The cells were allowed to form spheroids in a 5% CO_2_ humidified incubator at 37 °C for 24 h.

Then, a solution of 1.5 mg/mL of rat-tail collagen type-I (BD Biosciences, Oxford, UK) was prepared in EGM-2 medium and its pH was neutralized with 1 M NaOH. A first layer of this solution (20 μL) was deposited in each chamber of 8-well slides (iBidi, Martinsried, Germany) and incubated at 37 °C for about 30 min to allow it to set. Spheroids were collected from each well of a 96-well plate (one spheroid per well), into a 15 mL tube and centrifuged at 1500 rpm for 5 min to pellet them. The supernatant was removed, and the spheroids were washed once with 5 mL of phosphate-buffered saline (PBS) and resuspended in fresh collagen type-I solution (1.5 mg/mL as described above). A 20 μL drop containing the spheroids (with the exact number of spheroids per drop varying based on the distribution in the suspension) was placed on top of the first collagen layer, followed by another incubation at 37 °C for 1.0 to 1.5 h. Finally, 400 μL/well of M199 medium (supplemented with 2% FCS, 100 μg/mL penicillin/streptomycin, 50 ng/mL heparin, 25 μg/mL ECGS, and 2 mM L-glutamine, including inhibitors and stimulants when necessary) were added to the wells, and the cells were cultured at 37 °C for 40 h.

Subsequently, the collagen drops were washed 3 times with 400 μL/well of Hanks’ Balanced Salt Solution (HBSS, Gibco). Then, the spheroids embedded in the collagen matrix were fixed by adding 400 μL/well of 4% paraformaldehyde (*w*/*v*) in HBSS and leaving the slides at room temperature (RT) for 1 h. The fixed spheroids were washed 3 times with 400 μL/well PBS and stored in PBS at 4 °C in the dark until imaging. The spheroids were imaged with a Leica TCS SP8-X DLS confocal microscope (Leica Camera AG, Wetzlar, Germany) with a 10× objective lens, providing a total magnification of 100×. The methodology to quantify EC sprouting by digital image analysis was described in detail by Philippon et al. [41]. Briefly, confocal image Z-stacks were compiled in 2D projection using CI convert (CI Convert (LIF to Images and Images to LIF)—Software Cellularimaging, used versions 8.3 to 9.0, developed by the Cellular Imaging Core Facility, Amsterdam UMC, AMC, University of Amsterdam, Amsterdam, The Netherlands) while keeping the individual pixel values for all imaging channels. The converted pictures were uploaded into the QuPath image analysis program [42], available at https://qupath.github.io/ (accessed 28 September 2023), and the appropriate channels were employed for quantitative assessment of sprout area.

The area covered by migrated cells within the matrix, including areas that seemed either attached or detached from the core, underwent segmentation using a pixel classifier previously trained on a collection of representative images. The sprout area was distinguished from the core area of the spheroids. Each image was then reviewed and adjusted with a different trained pixel classifier when necessary [26,41,42] (Figure S2).

### 2.5. MTT Assay

To determine drug toxicity, cell viability and proliferation were evaluated by an MTT (3-[4,5-dimethylthiazol-2-yl]-2,5 diphenyl tetrazolium bromide) assay [43]. Shortly, HUVEC cells derived from different donors were cultured at an initial density of 5000 cells/well in a flat-bottom 96-well culture plate (VWR) pre-coated for 30 min with 0.2% gelatin/PBS. After 48 h, at 80% cell confluence, the test compounds were added in a final volume of 150 μL/ well at concentrations ranging from 0.1 to 10 μM for sunitinib and 1 to 20 μM for tofacitinib. For NIKi, cell viability tests were performed previously [26]. The plates were then incubated for an additional 24 h at 37 °C in a 5% CO_2_ humidified atmosphere. Blank wells (without cells) and wells with only cells (without compounds) were considered as controls (Ctrl). After 24 h, the media were replaced with 100 μL of media with 10% FCS and 50 μL of MTT solution (50 mg/mL). The plates were kept in the dark on an orbital shaker for 5 min, followed by 4 h incubation at 37 °C. Then, 150 μL of MTT solvent (acidified isopropanol) was added to each well, and the plates were gently mixed to ensure homogenization. MTT metabolization by viable cells into formazan product was measured at OD = 540 nm using a VersaMax™ microplate reader (Marshall Scientific, Hampton, NH, USA).

### 2.6. Statistical Analysis

Statistical significance was evaluated with Student’s two-tailed paired *t*-test for comparison between two groups. For comparisons involving more than 2 groups, one-way ANOVA and a non-parametric test (Kruskal–Wallis test) were used, followed by the original false discovery rate (FDR) method of Benjamini and Hochberg to adjust *p*-values. Discovery thresholds were set at q < 0.05 for multiple comparisons. Significant mean comparisons are annotated as *p*-values < 0.05 and non-significant (ns) ones as *p* > 0.05.

## 3. Results

### 3.1. Effectiveness and Toxicity of Small Molecule Compounds in HMEC-1 Cell Scratch Assay: Time-Dosing Profiles and Cellular Impact

**Figure 1:**
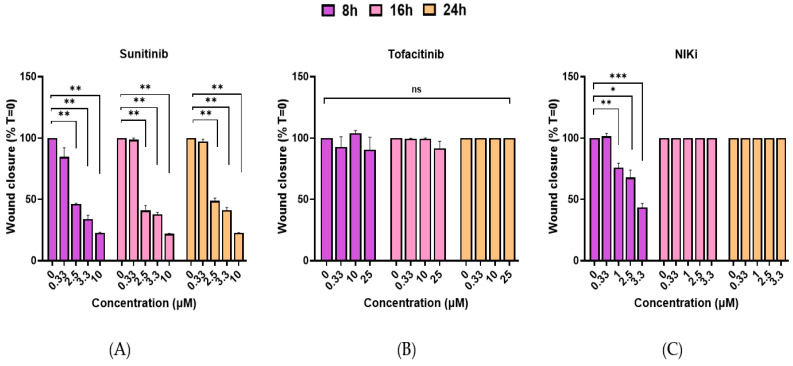
Effects of sunitinib, tofacitinib, and NIKi on HMEC-1 cell migration in scratch assay. HMEC-1 scratch wound analysis in the presence of (**A**) sunitinib (pan tyrosine kinase receptor inhibitor) at concentrations ranging from 0.33–10 µM. Analysis conducted after 8, 16, and 24 h drug incubations. (**B**) Tofacitinib (selective JAK1/3 inhibitor), concentration range: 0.33–10 µM, analysis performed after 4, 8, and 16 h drug incubations (with no stimulator added). (**C**) NIKi (NF-κB inducing kinase inhibitor) at concentrations ranging from 0.33–10 µM (in the presence of LIGHT (TNFSF14) to stimulate non-canonical NF-κB pathway. The % of migrating cells was calculated as the area covered by cells and is represented as an average of 3 independent experiments. Data represent mean ± SEM. *p*-values represent two-tailed distribution according to Student’s *t*-test (***: *p* ≤ 0.001, **: *p* ≤ 0.01, *: *p* ≤ 0.05 and ns: not significant).

**Figure 2:**
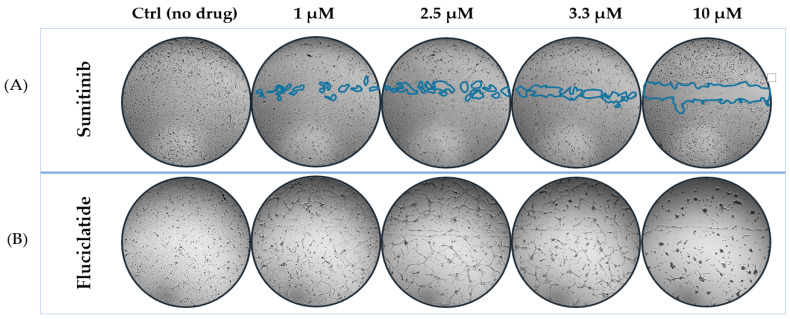
Scratch assay images of HMEC-1 cells treated with sunitinib and fluciclatide after 24 h of incubation. Representative images from scratch assay of HMEC-1 cells treated with (**A**) sunitinib and (**B**) fluciclatide following 24 h of drug incubation. The images were captured on an automated platform using a Leica DMI3000B microscope (Leica, Rijswijk, The Netherlands) and a Pulnix RMC1327GE camera (Takex Europe, Hampshire, UK) under control of Universal Grab 6.3 software (DCILabs, Keerbergen, Belgium).

The scratch assay was used to evaluate the impact of various small molecules on the ability of HMEC-1 cells to close a scratch in the cultured monolayer of EC, which is indicative of their potential to inhibit cell migration and “wound healing”—a key aspect of angiogenesis. The time-dosing effects of multiple candidate anti-angiogenic drugs in the scratch assay with HMEC-1 cells are presented in Figure 1. At 2.5 μM and 3.3 μM, sunitinib, as a positive control, significantly inhibited scratch closure by approximately 40% and 52%, respectively, after 8 h. This effect was maintained at 16 and 24 h without further enhancement (Figure 1A). However, at 10 μM sunitinib, a maximum of ∼75% scratch closure inhibition was achieved, and this level of inhibition was sustained throughout the 24 h incubation period. We examined using an MTT assay whether part of this effect may have been due to drug-induced cellular toxicity. A 24 h period of exposure to sunitinib showed an IC50 of 8.5 μM (Figure S3A), meaning that, at this concentration, 50% of the cells’ viability was inhibited. For the JAKi tofacitinib, no significant inhibitory effects on scratch closure were observed for concentrations up to 25 μM and drug exposure times up to 24 h (Figure 1B). No toxic effects of tofacitinib were observed in MTT assays on EC and drug exposure times of 24 h (Figure S3B). A transient inhibition of scratch closure was noted, with NIKi showing 24% inhibition at 2.5 μM, 32% at 3.3 μM, and a peak inhibition of nearly 60% at a concentration of 10 μM after 8 h of incubation (Figure 1C). The inhibitory effect was not maintained after prolonged cultures for 16 and 24 h. Fluciclatide unexpectedly and markedly induced morphological changes to the HMEC-1 cultures over 24 h (Figure 2B). At 10 µM, the drug appeared highly toxic, and widespread cell death and tube-like structures were noted. Figure 2A,B show representative images of HMEC-1 cell morphology and scratch areas after 24 h of incubation with different concentrations of sunitinib (Figure 2A) and fluciclatide (Figure 2B). While significant morphological alterations were evident with fluciclatide on HMEC-1 cells, which are commonly used as an alternative to HUVECs, no effects on HUVECs were observed (Figure S4A,B).

### 3.2. Effects of Small-Molecule Compounds in 3D Spheroid Model of General Angiogenesis with HUVEC and NHDF Stimulated with Growth Factors

**Figure 3:**
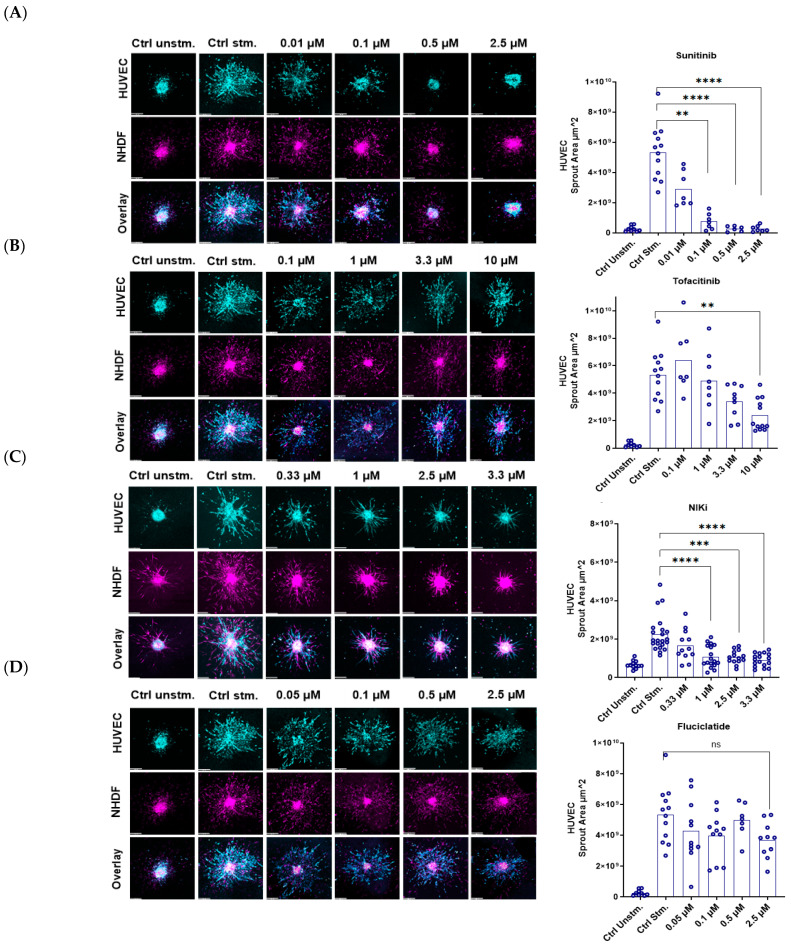
Inhibition of endothelial cell sprouting in 3D spheroid-based angiogenesis model treated with sunitinib, tofacitinib, NIKi, and fluciclatide. Representative confocal images (10× magnification) of 3D spheroid-based angiogenesis model composed of HUVEC depicted (in cyan) and NHDF cells (in magenta) upon stimulation with the growth factors VEGF/bFGF. Inhibition of EC sprouting was monitored after 40 h of incubation with: (**A**) sunitinib, (**B**) tofacitinib, (**C**) NIKi, and (**D**) fluciclatide. Sprout formation of HUVEC was quantified by measuring the total sprout area of each spheroid, defined by training pixel classifiers in QuPath. Data represent the means of 3 independent experiments. Statistical analysis was conducted using one-way ANOVA, a non-parametric test, followed by the original FDR method of Benjamini and Hochberg with *p*-values: ****: *p* ≤ 0.0001, ***: 0.0001 < *p* ≤ 0.001, **: 0.001 < *p* ≤ 0.01, ns: not significant.

This experiment evaluated the anti-angiogenic effects of drugs using a 3D model with HUVEC and NHDF, which were stimulated with the growth factors VEGF and bFGF (GF) to mimic the context of angiogenesis. EC sprouting inhibition was quantified to indicate the potential of the inhibitors to block angiogenesis within this model. Representative confocal pictures of the spheroids and their respective EC sprouting areas for each treatment are shown in Figure 3. The addition of GF resulted in a significant increase in EC sprout area compared to the unstimulated condition. In comparison with the EC sprouting area in the presence of GF only, sunitinib demonstrated a significant inhibitory effect. At concentrations of 0.1 μM and 2.5 μM, sunitinib markedly blocked sprout formation by ~85% (*p* < 0.01) and ~94% (*p* < 0.0001), respectively (Figure 3A). Tofacitinib exhibited modest inhibitory effects at 3.3 µM (~35%, p: ns) and 10 µM (~55%, *p* < 0.01) concentrations (Figure 3B). NIKi showed inhibitory effects at concentrations of 1.0 μM (~66%, *p* < 0.0001) and 2.5 μM (~63%, *p* < 0.001) (Figure 3D). Although the inhibition by NIKi at 2.5 μM was slightly lower than that at 1.0 μM, the overall trend indicated a concentration-dependent effect across the tested range of 0.33 μM, 1.0 μM, 2.5 μM, and 3.3 μM. At 3.3 μM, NIKi was beyond the IC50 range and showed ~73% (*p* < 0.0001) inhibition. Fluciclatide marginally inhibited EC sprouting at 2.5 μM (26.5%, p: ns), and no significant inhibition was observed at concentrations of 0.05, 0.1, or 0.5 uM (Figure 3C).

### 3.3. Effects of Small-Molecule Compounds on the 3D Spheroid Model of RA Synovial Angiogenesis

**Figure 4:**
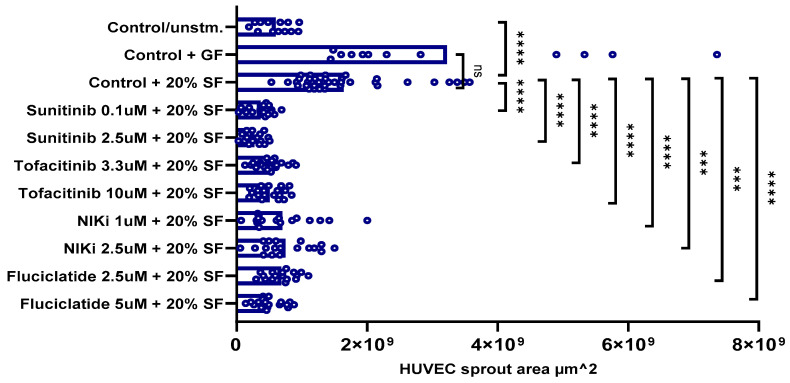
Effects of inhibitors on HUVEC sprouting in a 3D spheroid-based model of RA synovial angiogenesis. Drug incubations: 40 h. Statistical significance was determined using one-way ANOVA, a non-parametric test, followed by the original FDR method of Benjamini and Hochberg with *p*-values: ****: *p* ≤ 0.0001, ***: 0.0001 < *p* ≤ 0.001, and ns: not significant.

This 3D spheroid model was designed to evaluate the anti-angiogenic effects of novel therapeutic compounds in a more complex synovial microenvironment by incorporating HUVEC and RAFLS cultured in the presence of RA SF. Similar readouts were used as in the general angiogenesis 3D spheroid model. In this setup, the EC sprouting area under SF-stimulated conditions was significantly increased compared to the unstimulated conditions (Figure 4). However, the SF-stimulated sprouting area was lower than when the model was stimulated with GF (Figure 3). Building on previous 3D spheroid experiments, we opted for two concentrations to test for each drug: 0.1 and 2.5 μM for sunitinib, 3.3 and 10 μM for tofacitinib, 1.0 and 2.5 μM for NIKi, and 2.5 and 5.0 μM for fluciclatide. In comparison to the SF stimulation only, our study revealed significant inhibitory effects across all treatments. Specifically, 0.1 μM of sunitinib inhibited ~77% of EC sprouting (*p* < 0.0001), while a higher concentration of 2.5 μM sunitinib demonstrated a slightly increased inhibition (~83%, *p* < 0.0001). Tofacitinib exhibited ~69% inhibition (*p* < 0.0001) at both concentrations of 3.3 μM and 10 μM. NIKi demonstrated inhibition rates of ~57% (*p* < 0.0001) and ~53% (*p* < 0.001) at 1.0 μM and 2.5 μM, respectively (Figure 4). Fluciclatide showed dose-dependent inhibition with 2.5 μM and 5.0 μM doses, suppressing sprouting by ~58% (*p* < 0.001) and ~70% (*p* < 0.0001), respectively.

## 4. Discussion

Our comparative analysis unveiled distinct patterns of anti-angiogenic effects among the compounds (i.e., tofacitinib, NIKi, and fluciclatide, alongside sunitinib as a positive control) in both complementary in vitro models, i.e., the scratch and 3D culture assays. While the scratch assay can serve as a straightforward 2D/monolayer drug-testing system using either immortalized or primary EC [40], the 3D spheroid model system incorporating EC and RAFLS more closely represents the angiogenesis-relevant cellular composition and 3D microenvironment conditions within the RA synovium [26]. The inhibitory effects of the drugs tested reflect differences in their mechanisms of action and efficacy. Tofacitinib and NIKi both demonstrated moderate suppression of EC sprouting and migration, but their effects were less potent than those of sunitinib, which had the strongest anti-angiogenic inhibition. Fluciclatide altered EC morphology rather than migration. In the spheroid models, it showed its inhibitory effect at ≥2.5 μM, suggesting that a safe dose for diagnostic and theranostic use should be under 2.5 μM.

In this study, sunitinib as a pan-kinase inhibitor was utilized not only as a positive control to confirm the anti-angiogenic response in our models, but also to investigate its impact within the RA microenvironment. It exhibited marked and sustained inhibitory effects on EC migration in the scratch assay and on EC sprouting in the two different 3D models. Sunitinib primarily targets EC, which suggests its utility in contexts beyond oncology, including inflammatory diseases like RA, where angiogenesis contributes to disease pathology [44]. However, despite its promising effects, due to its clinical side-effects (e.g., hypertension, fatigue) [45], it may not be a viable treatment for RA. Sunitinib impairs VEGFRs’ critical signaling pathways needed for cell migration and survival, resulting in a prolonged inhibition of EC migration in scratch assays [4]. Sunitinib also inhibits the VEGF-induced tyrosine phosphorylation and cleavage of VE-cadherin in EC, which hampers the growth of new blood vessels [46]. Additionally, sunitinib visually inhibited fibroblast proliferation in the 3D model, suggesting its potential to suppress both angiogenesis and fibroblast-driven inflammation in RA. This aligns with findings from a murine arthritis model, where sunitinib significantly reduced arthritis severity and vascular density in the synovial membrane [28].

Likewise, it is important to carefully assess the dose-dependent effects of tofacitinib and NIKi on both vascular density and arthritis severity. At present, JAK inhibitors are well-established in RA treatment for their immunomodulatory properties [47], but their impact on angiogenic processes was less investigated. Tofacitinib, one of the first clinically applied JAKis, displayed significant anti-angiogenic effects in an experimental arthritis model and also in RA patients after 1 year [30,48]. Apart from its immunomodulatory activity, tofacitinib also reduced synovial vascular density, VEGF, and Ang-2 levels in the collagen-induced arthritis (CIA) model, validating its previously established anti-angiogenic function in vitro [30]. Based on these findings, it was postulated that tofacitinib can affect all stages of vascular network from migration to tube formation [30]. Although VEGFR2 signaling is typically regulated by JAK2, the study indicated that tofacitinib, a selective JAK1/3 inhibitor, also affected angiogenesis by disrupting the VEGF signaling pathway [30]. Another study in a 2D coculture system of RA-FLS and HUVECs also showed that tofacitinib inhibits the migration, proliferation, and tube formation of ECs and reduces the production of VEGF [49]. Notably, tofacitinib also exerts anti-angiogenic effects by decreasing the production of IL-6, VEGF, bFGF, epidermal (EGF), and placental (PlGF) growth factors in RA patients after one year of therapy [48]. However, in the scratch assay, tofacitinib had no apparent effects on HMEC-1 cell migration at concentrations up to 10 μM, which is well above the clinically achievable plasma concentrations of this drug [50]. This discrepancy could be due to the absence of specific stimuli, such as interleukins, that activate the JAK1/3 pathway, which tofacitinib primarily targets [30,51]. Moreover, the scratch assay might not be sensitive enough to detect subtle effects of tofacitinib, whereas other assays like trans-well migration or proliferation assays might be more suitable for assessing tofacitinib’s impact on HUVEC cells. Our findings in the spheroid models align with previous observations [49], as tofacitinib concentrations ranging from 3.3 to 10 μM exhibited clear inhibitory effects on EC sprouting. This may highlight the role of fibroblasts in the production of proangiogenic components activating the JAK/STAT pathway [52]. Whether these observations also apply to other (more selective) JAK inhibitors, like upadacitinib [53], should be investigated further in future studies.

Rather than aiming for broad kinase inhibitory activity, like for sunitinib, which can disrupt EC function and impair normal vascular integrity [45], more selective kinase inhibitors (i.e., NIKi) may specifically target certain pathological processes, potentially reducing side-effects. In the scratch assay, NIKi affected cell migration, though the effects were limited in time. This may have been due to the competitive effects of ATP and/or drug efflux systems in HMEC-1 cells. Since NIK is continuously degraded and does not accumulate under homeostatic conditions, the effects of NIKi might be reversible once the inhibitor is no longer present. Taken together, NIKi deserves further (pre)clinical evaluation as an angiogenesis inhibitor drug in RA.

While VEGF signaling was not directly evaluated in our study, recent research in a model of colorectal cancer angiogenesis suggests that NIKi may limit angiogenesis by targeting the noncanonical NF-κB pathway, which can act as a compensatory mechanism when VEGF signaling is compromised [54]. Also, synergy between bevacizumab (an anti-VEGF antibody) and NIKi was demonstrated, since their combination significantly reduced VEGF- and bFGF-induced sprouting [54]. In another study, angiogenesis was reduced in the aortic assay in both antigen-induced arthritis (AIA) and B16 melanoma models in NIK-deficient mice, while VEGF-induced angiogenesis was unchanged [55]. Consequently, targeting non-canonical NF-κB signaling may selectively inhibit pathological angiogenesis in disorders such as RA and cancer, possibly overcoming resistance to anti-VEGF therapy [55].

The more prominent effects of tofacitinib and NIKi in the spheroid model underline the importance of mimicking multicellular interactions in a 3D microenvironment where cytokines and pro-inflammatory molecules are being produced. Cytokines like tumor necrosis factor (TNF)-α, interleukin (IL)-6, and IL-1β, which are secreted by EC and fibroblasts and are also present in the synovial fluid, contribute to the inflammatory microenvironment. These cytokines may impact ECs and fibroblasts through signaling pathways such as JAK1/3 and NF-κB, which are crucial for EC activation and angiogenesis [26]. This interplay influences angiogenesis directly on the EC or indirectly through interactions with the fibroblasts, which may boost the effects of the inhibitors. These models better reflect the inflammatory environment compared to the simpler angiogenesis assays, offering a more comprehensive understanding of their efficacy and mechanisms of action.

It is worth mentioning that a recent study showed how soluble factors from T memory cells can activate the ECs, with the NF-κB and JAK/STAT pathways being central to this process [32]. It was found that tofacitinib reduces IL-6 production of ECs and, when combined with NIKi, shows additive therapeutic effects [32]. Taken together, these findings show the therapeutic potential of combining these selective inhibitors for more effective treatment of RA [32]. Fluciclatide was included as potential agent for monitoring by PET/CT imaging, since [^18^F]-fluciclatide as a PET-imaging tool has shown promising results to monitor angiogenesis in rodent and human tumors, and may also be valuable for monitoring RA angiogenesis in vivo and/or evaluation of treatment effects. It exerts its effects through targeting integrin α_v_β_3_/α_v_β_5_ [56], and the potential threshold dosage level of biologic activity is currently unknown. Fluciclatide applied to HMEC-1 unexpectedly triggered dose-dependent morphological changes resembling vascular network formation. These effects were previously reported for in vitro adherent HMEC-1 cells when cultured in media supplemented with extracellular factors, including VEGF, IGF, EGF or FCS [27]. These morphological changes may have been due to the alteration of integrin functions, as they are essential for signaling and cell adhesion. Targeting integrins may interfere with cell attachment to cell substrates containing collagen and/or gelatin and impact interactions between cells, thereby contributing to the creation of the observed network. Whether this represents a potential side effect of fluciclatide on EC in vivo remains to be established. The inhibition of EC sprouting by fluciclatide in the 3D model at a concentration of 2.5 μM and higher suggests that its primary mechanism of action involves targeting integrins on these cells. No (significant) biologic activity was observed below 2.5 μM, which supports safe application as a diagnostic/theranostic probe at a microdose for PET/CT monitoring of the effects of anti-angiogenic drugs.

A potential limitation of the current study is the use of different EC types, including primary HUVECs and immortalized HMEC-1 cells. This may have caused variability in the results because of potential differences in their cellular responses. Nevertheless, this can also be seen as a strength, as we have evaluated the effect of the inhibitors both in cell lines and in primary EC.

## 5. Conclusions

The current study demonstrates the robust anti-angiogenic properties of tofacitinib and NIKi, which may hold promise as therapeutic approach in RA treatment. Tofacitinib may indirectly influence angiogenesis through the regulation of VEGF/VEGFR signaling pathways, and NIKi may block non-canonical NF-κB signaling, which acts as a compensatory pathway when VEGF signaling is compromised. Our results also provide a safe microdosage cutoff threshold for fluciclatide, to be potentially used when radiolabeled as a diagnostic and theranostic probe in PET imaging to track the effects of anti-angiogenic medications in RA and beyond. Together, these findings not only advance our understanding of the complex regulation of VEGF/VEGFR signaling, but also contribute to research efforts ultimately aimed at the development of targeted therapeutic strategies applicable to inflammatory disorders like RA and other angiogenesis-driven disorders.

## Data Availability

Data are contained within the article or Supplementary Material.

## Supplementary Materials

The following supporting information can be downloaded at: https://www.mdpi.com/article/10.3390/cells14020102/s1, Figure S1: Molecular structures of (A) sunitinib, (B) tofacitinib, (C) NIKi, and (D) fluciclatide.; Figure S2: Schematic overview of the experimental steps for spheroid assay. Figure S3: MTT (cytotoxicity) dose–response curve of HUVEC to (A) sunitinib and (B) tofacitinib. Figure S4: Differential effects of fluciclatide on HMEC-1 and HUVEC scratch assay at various time points.
