# Supplementary material for: Unveiling the Anti-Angiogenic Potential of Small-Molecule (Kinase) Inhibitors for Application in Rheumatoid Arthritis
Source: Cells. 2025 Jan 11;14(2):102. doi: 10.3390/cells14020102 (PMC11764070; doi:10.3390/cells14020102)
Supplement: Supplementary file 1 [file cells-14-00102-s001.zip › cells-3398288-supplementary.pdf]

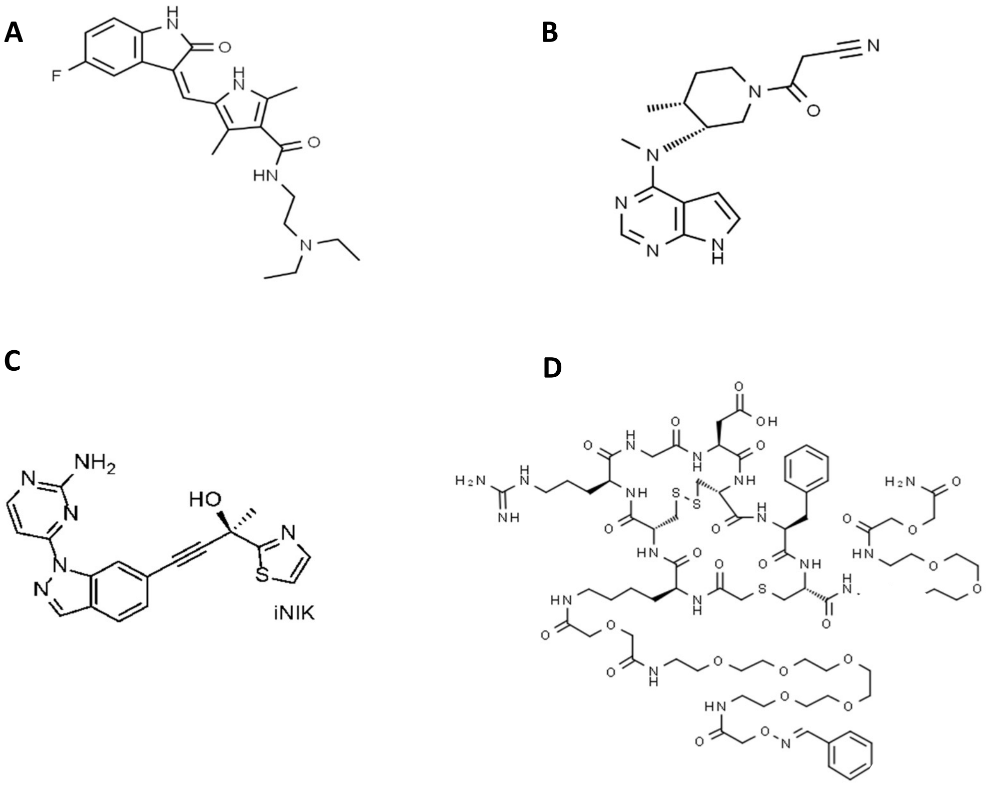

Figure S1. *Molecular structures of (A) Sunitinib, (B) Tofacitinib, (C)NIKi , and (D) Fluciclatide. A-C: Molecular structures were obtained from ChemSpider (http://www.chemspider.com/Chemical-Structure.1906.html) on April 17, 2024. Chemical Structure Identifiers (CSID) used: CSID:4486264, CSID:8102425, CSID:30790871. D: Molecular structure obtained from the supplementary file of the article with DOI: 10.3389/fimmu.2022.860327.*

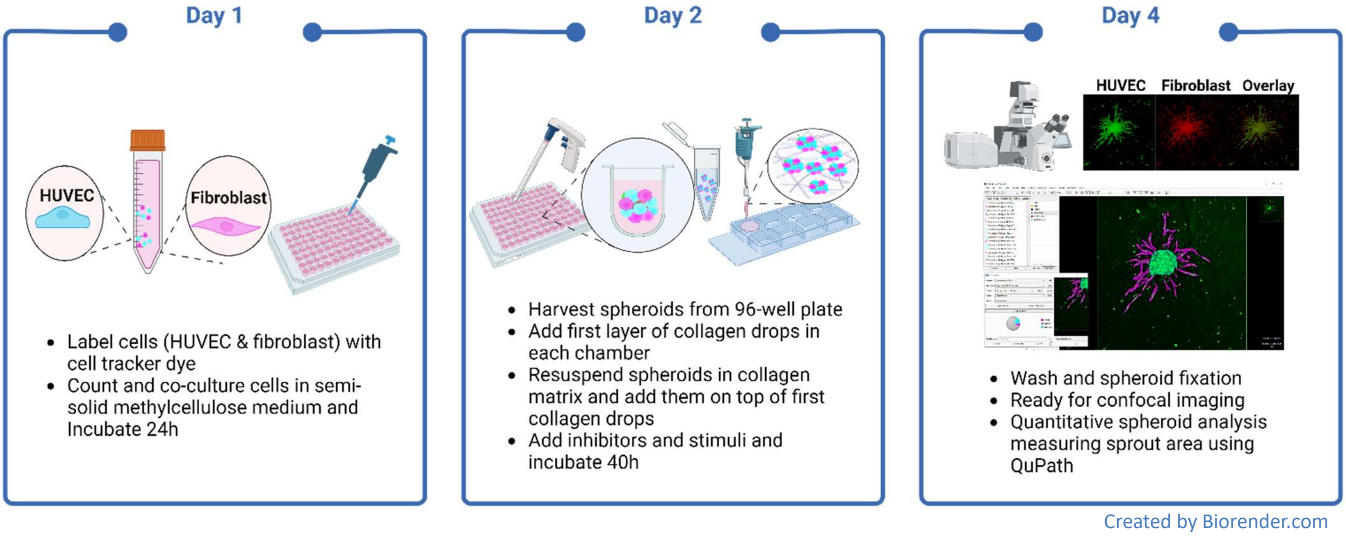

Figure S2. *Schematic overview of the experimental steps for spheroid assay. The experimental procedure involved labeling and co-culturing cells overnight to form spheroids in 96-well plates with methocel solution. These spheroids were then embedded in collagen on slides. After setting of the collagen in 37 °C incubator, medium with supplements, including stimulants (VEGF/bFGF) or inhibitors, was added. Following 40 hours of incubation, sprout growth was assessed. Sprouts were then imaged and analyzed using confocal microscopy and QuPath software.*

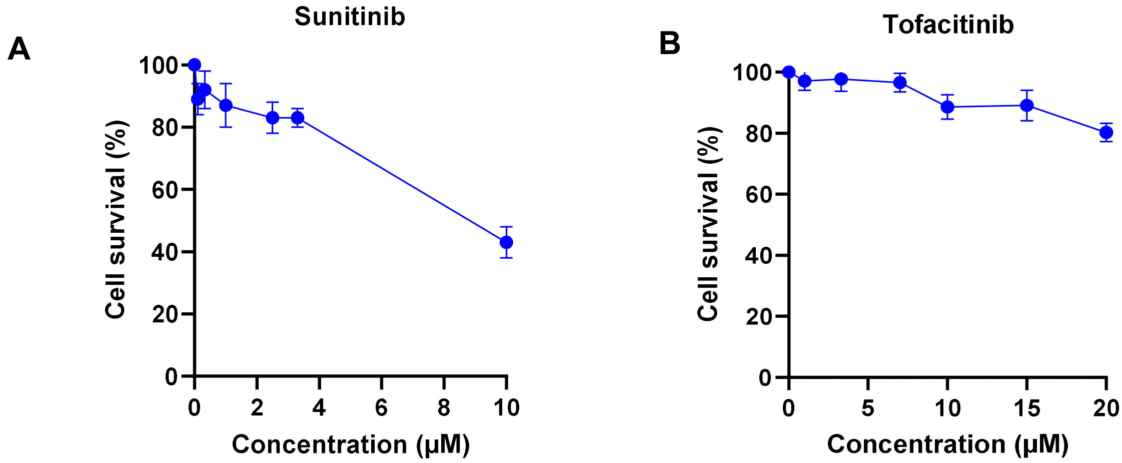

Figure S3. *MTT (cytotoxicity) dose response curve of HUVEC to (A) Sunitinib and (B) Tofacitinib. Drug exposure: 24 hours. Experiments were performed in quadruplicate, and the average of 3 separate experiments are shown expressed as percentage of Ctrl (set to 100%) and mean ± standard deviation (SD). Data represents the Mean ± SEM of 3 independent experiments.*

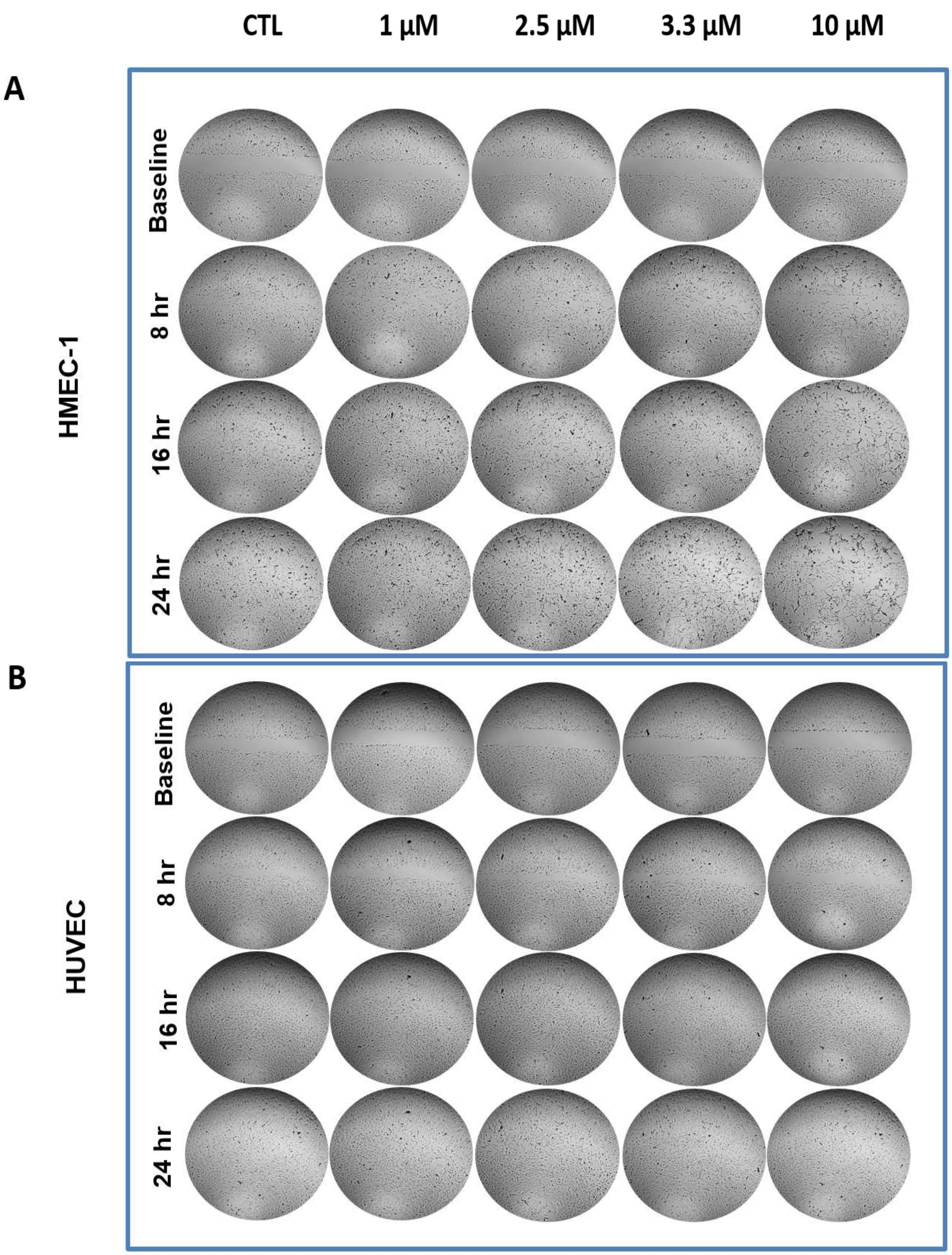

Figure S4. *Differential effects of fluciclatide on HMEC-1 and HUVEC scratch assay at various time points. Representative images from the scratch assay of HMEC-1 (A) and HUVEC (B) cells treated with different concentration of fluciclatide (1-10 µM) at different time points: 8, 16, and 24 hours.*
